# Hybrid management of a spontaneous ilio-iliac arteriovenous fistula: a case report

**DOI:** 10.1186/1752-1947-5-401

**Published:** 2011-08-22

**Authors:** Gavin C O'Brien, Colm Murphy, Zenia Martin, Naseem Haider, Mary P Colgan, Dermot Moore, Prakash Madhavan, Sean M O'Neill

**Affiliations:** 1 St James' Hospital Vascular Department, St James' Hospital, Dublin 6, Ireland

## Abstract

**Introduction:**

Spontaneous iliac arteriovenous fistulae are a rare clinical entity. Such localized fistulation is usually a result of penetrating traumatic or iatrogenic injury. Clinical presentation can vary greatly but commonly includes back pain, high-output congestive cardiac failure and the presence of an abdominal bruit. Diagnosis, therefore, is often incidental or delayed.

**Case presentation:**

We report a case of a spontaneous ilio-iliac arteriovenous fistula in a 68-year-old Caucasian man detected following presentation with unilateral claudication and congestive cardiac failure. Following computed tomography evaluation, the fistula was successfully treated with a combined endovascular (aorto-uni-iliac device) and open (femoro-femoral crossover) approach.

**Conclusion:**

Endovascular surgery has revolutionized the management of such fistulae and we report an interesting case of a high-output iliac arteriovenous fistulae successfully treated with a hybrid vascular approach.

## Introduction

Spontaneous iliac arteriovenous fistulae (AVF) are a rare clinical entity. Such localized fistulation is usually a result of penetrating traumatic or iatrogenic injury [1]. Clinical presentation can vary greatly but commonly includes back pain, high-output congestive cardiac failure (CCF) and the presence of an abdominal bruit. Diagnosis, therefore, is often incidental or delayed [2].

### Case presentation

**Figure 1:**
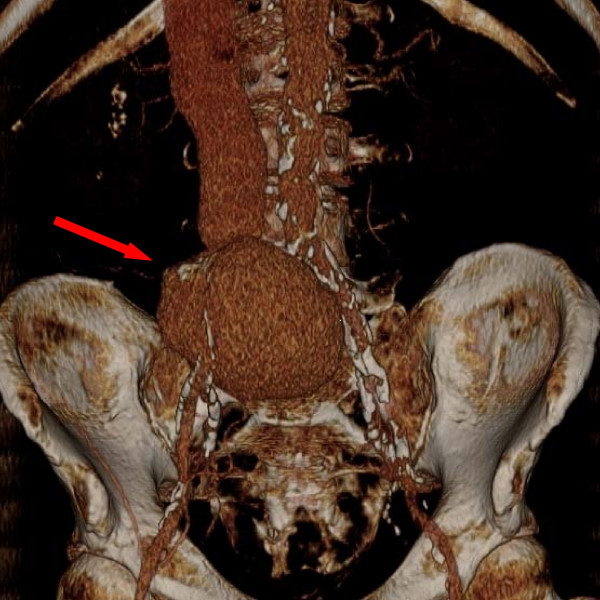
**Preoperative CT**. Preoperative contrast-enhanced CT 3-D reconstruction with arrow demonstrating the 8 cm ilio-iliac AV fistula originating from the proximal right CIA.

A 68-year-old Caucasian man was referred to our out-patients clinic following the incidental discovery of a 6 × 8 cm distended iliac vessel whilst having an ultrasound for surveillance of liver cirrhosis. His clinical history revealed a progressive history of right flank pain, worsening right leg claudication and a persistently cold sensation in his right foot. He also complained of progressive breathlessness on exertion, clinically suggestive of deteriorating CCF. There was no history of trauma or previous surgery. Ankle-brachial pressure measurements revealed a reduced index of 0.76 on the right, and a normal index of 1.35 on the left with corresponding toe pressures of 55 mmHg and 143 mmHg respectively. A contrast enhanced computed tomography (CT) scan showed an isolated right common iliac artery (CIA) to right common iliac vein AVF (Figure 1). His aorta was normal in caliber, measuring 19 mm at the aortic bifurcation. The aneurysmal segment began immediately distal to the aortic bifurcation at the origin of the right CIA, with no normal segment of CIA evident. The aneurysm measured 9.4 cm in maximal diameter and extended to within 17 mm of the right iliac bifurcation. It was impossible to differentiate the arterial wall from the venous wall in the aneurysmal segment on either ultrasound or CT scans. His inferior vena cava (IVC) was grossly distended with a uniform diameter of 36 mm in its full course.

Pre-operative discussions focused on finding an endovascular strategy to solve the problem. As no normal caliber proximal right CIA existed, an isolated iliac covered stent was impossible as no proximal sealing zone existed. This required sealing a proximal stent in his aorta. Bifurcated endografts have been used to seal ilio-iliac fistulae previously [1]. The aortic bifurcation diameter was 19 mm in this case and although some devices have reported success negotiating smaller aortic bifurcations [2], we felt an aorto-bi-iliac graft would be in danger of being compressed at the bifurcation with a high risk of occlusion. As a result, a hybrid approach with deployment of an aorto-uni-iliac (AUI) device followed by a femoro-femoral bypass was planned. Our patient was operated upon in our endovascular suite (Siemens) under spinal anesthesia. Both common femoral arteries (CFA) were controlled and cannulated with 6Fr sheaths (Johnston & Johnston). His aorta was cannulated with a 0.035 wire Bentson wire (Cook Medical, Bloomington, IN, USA) via each CFA with the assistance of an angled catheter (Kumpe, Cook Medical). A diagnostic flush pigtail catheter was placed in his aorta via the contralateral limb. A stiff 0.035 Amplatz wire (Amplatz Super Stiff, Boston Scientific) was exchanged as access to the ipsilateral limb. Following an angiogram to confirm renal anatomy, an aorto-uni-iliac device (Zenith Renu, Cook Medical) was deployed from immediately below his renal arteries to his mid right external iliac artery, thus occluding his right internal iliac artery. A 14 × 10 mm Amplatzer occlusion device (AGA Medical Corp, MN, USA) was deployed via the contralateral limb to his proximal left CIA. A completion angiogram confirmed exclusion of the ilio-iliac AVF. A right to left femoro-femoral bypass with 8 mm polytetrafluoroethylene (PTFE) was performed to re-establish flow to his left leg as well as providing left internal iliac perfusion.

**Figure 2:**
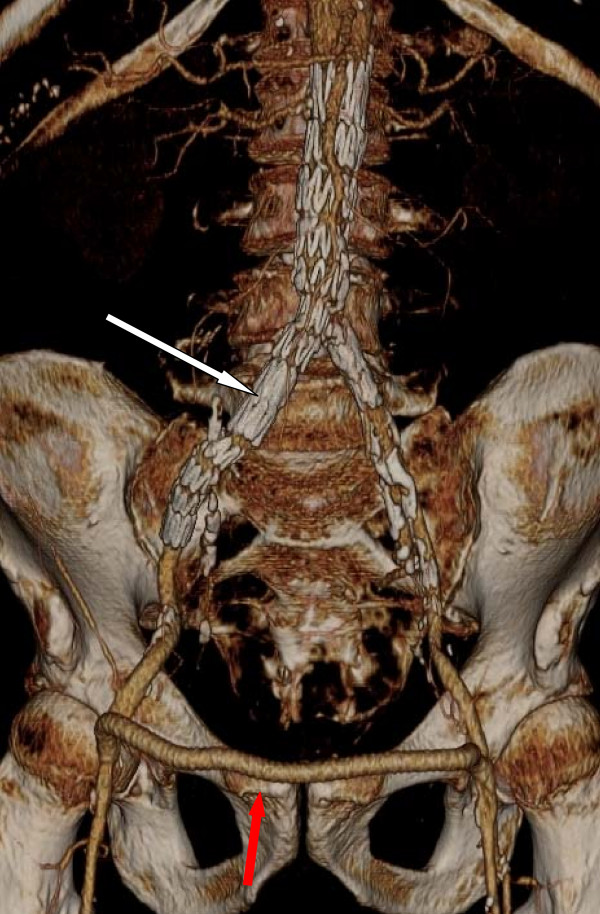
**Postoperative CT**. Postoperative CT 3-D reconstruction showing endovascular exclusion of the AV fistula with an aorto-uni-iliac graft (white arrow) and a functioning right to left femoro-femoral cross-over bypass (red arrow).

On the first postoperative day, the mass was no longer pulsatile and the machinery-like murmur in the right iliac fossa was absent. A CT scan on postoperative day two confirmed a patent AUI endovascular graft and patent femoro-femoral bypass, as well as confirming exclusion of contrast from the right iliocaval system (Figure 2). His right foot no longer felt cool and the postoperative ankle brachial index (ABI) confirmed an index of 1.05 on the right and 0.95 on the left, with corresponding toe pressures of 119 mmHg and 117 mmHg respectively. He was discharged after seven days without complication. At a clinic one month later, his claudication had resolved completely and he no longer had symptoms suggestive of CCF. A duplex ultrasound confirmed exclusion of arterial flow from the iliocaval vessels.

## Discussion

Penetrating traumatic injury remains the most common cause of abdominal and pelvic arteriovenous fistulae [3]. This trauma may be malicious but is frequently iatrogenic, commonly occurring after lumbosacral laminectomy whereupon penetration of the anterior longitudinal ligament by dissecting instruments can injure the aorta, IVC or iliac vessels, depending on the level of the laminectomy [4]. Linton and White first reported such a case in 1945 [5]. Case reports also exist of fistulation following aortic aneurysm surgery and even after laparoscopic appendectomy [6]. Post-traumatic AVFs may present many years after the initial injury, some reporting a traumatic history as distant as 30 to 52 years previously [7]. In such cases, the remote injury in combination with the diverse and subtle modes of presentation can result in delayed or overlooked diagnosis of the fistula. Typical symptoms include back pain (70%) and progressive sequelae from high output CCF (such as orthopnoea, edema and fatigue) [4]. An abdominal bruit is commonly demonstrable (80%). Our patient reported unilateral, progressive leg claudication, confirmed with unilaterally reduced ABI and toe pressure readings. This reflected a steal phenomenon which was subsequently fully reversed following exclusion of the AVF. To the best of our knowledge, this interesting phenomenon has not previously been described.

Treatment of iliac AVFs in the open vascular surgical era was fraught with danger, with reported surgical mortality rates of 9-34%. Operative blood loss of six liters was common [8]. Acute presentations with spontaneous ruptures are thankfully rare (less than 4% of ruptured aneurysms [9]) but carry a significantly higher mortality rate.

## Conclusion

The evolution of endovascular surgery has led to a paradigm shift in the approach to managing aneurysmal disease with a concomitant vast reduction in mortality and morbidity rates. The first endovascular exclusion of an iliocaval fistula appeared in 1995 [10]. We add to the literature this report of a successful endovascular exclusion of a giant, spontaneous, ilio-iliac AVF. Our case demonstrates several aspects of modern day vascular surgery such as the importance of preoperative imaging and planning, as well as the emerging role for a hybrid endovascular and open surgical approach to minimize operative morbidity as well as optimize long-term success for such complex vascular pathology.

## Consent

Written informed consent was obtained from the patient for publication of this case report and any accompanying images. A copy of the written consent is available for review by the Editor-in-Chief of this journal.

## Competing interests

The authors declare that they have no competing interests.

## Authors' contributions

GOB was the main author, treated our patient, researched the topic and coordinated the editing of the paper. CM & ZM were major contributors to researching, writing and editing the paper. NH, MPC, PM, DM & SON were all involved in making treatment decisions regarding our patient as well as reading, making editorial suggestions and approving the final manuscript. All authors read and approved the final manuscript.
